# Altered gait strategies show inconsistent medial compartment unloading in varus medial knee osteoarthritis awaiting high tibial osteotomy

**DOI:** 10.1016/j.clinbiomech.2026.106777

**Published:** 2026-06

**Authors:** Jake Bowd, Sam Van Rossom, David W. Elson, Chris Wilson, Ilse Jonkers, Cathy Holt, Gemma M. Whatling

**Affiliations:** a Cardiff School of Engineering, College of Physical Sciences and Engineering, Cardiff University, Cardiff, UK; b Arthritis UK Biomechanics and Bioengineering Research Centre, Cardiff University, Cardiff, UK; c Human Movement Biomechanics Research Group, Department of Movement Science, KU Leuven, Leuven, Belgium; d Queens Elizabeth Hospital, Gateshead, UK; e University Hospital of Wales, Cardiff, UK; f Sport, Exercise, and Rehabilitation Sciences, School of Natural Sciences, University of Kent

## Abstract

**Background:**

Medial knee osteoarthritis is increasingly diagnosed in younger adults who are often unsuitable for joint replacement. High tibial osteotomy corrects varus malalignment but is invasive. Gait retraining is a low-cost, non-surgical option to reduce medial tibiofemoral loading, but its effects in varus deformity are unclear. We quantified the immediate biomechanical effects of short-term gait modifications on internal tibiofemoral loading.

**Methods:**

Twenty-nine patients (30 knees) with medial knee osteoarthritis scheduled for high tibial osteotomy performed three modified gaits: toe out, wide base, and medial thrust. Motion capture and musculoskeletal modelling estimated internal tibiofemoral joint forces in this pre- high tibial osteotomy, varus-aligned cohort.

**Findings:**

Toe out increased medial loading in early stance but reduced it in late stance. Wide base increased medial and lateral forces early, then reduced medial loading later with compensatory lateral increases. Medial thrust was difficult: only 20/30 knees achieved the target reduction in maximum knee adduction angle, and successful trials still increased early-stance loading. Overall effects were modest, phase-specific, and inconsistent.

**Interpretation:**

Generic gait modifications produced small, phase-dependent changes in internal tibiofemoral loading, with early-stance increases, late-stance reductions, and occasional compensatory lateral loading. Longer-term, individualised retraining incorporating symptoms is needed to determine net clinical benefit.

## Highlights

• Altered gait styles produced phase-specific changes in medial knee loading.
• Toe out and wide stance gait reduced medial loading in late, not early, stance.
• Medial thrust gait was difficult to perform and increased early stance loading.
• Individualised, longer-term gait retraining may better support knee osteoarthritis care.

## 1 Introduction

Patients with medial knee osteoarthritis (mKOA) and varus knee alignment can experience a treatment gap, as they may not be appropriate candidates for total knee arthroplasty; this is particularly relevant in younger adults but can also apply across adulthood. If left untreated, varus alignment increases medial compartment loading and is associated with structural progression in varus mKOA (Palmer et al., 2020). In symptomatic patients with varus malalignment, high tibial osteotomy (HTO) may be offered to correct alignment and offload the medial compartment.

Gait retraining is a low-cost, non-surgical strategy intended to modify lower-limb mechanics and reduce medial tibiofemoral loading and may be clinically relevant as a pre-surgical adjunct while patients await HTO (Bowd et al., 2019; Simic et al., 2011). Prior work has evaluated gait modifications such as toe out, wide stance walking, and medial thrust in healthy participants and in individuals with mKOA, largely without substantial varus deformity or using simulated malalignment (Favre et al., 2016; Gerbrands et al., 2014; Legrand et al., 2021; Ogaya et al., 2015; Schache et al., 2008). However, individuals with clinically relevant varus deformity represent an important in vivo group in which medial compartment loading is already elevated and responses to gait retraining may differ. Despite this, there remains limited evidence on whether commonly reported gait strategies meaningfully alter internal knee joint loading in varus-aligned patients awaiting HTO.

Toe out gait is proposed to reduce medial loading by shifting the ground reaction force (GRF) vector closer to the knee joint centre and reducing the frontal-plane moment arm (Gerbrands et al., 2014). Wide stance gait may lateralise the centre of pressure and similarly reduce the frontal-plane moment arm (Fregly et al., 2008). Medial thrust aims to reduce varus alignment during stance and thereby decrease the frontal-plane lever arm (Gerbrands et al., 2017). While these strategies may reduce surrogate measures such as the knee adduction moment in some populations, their effects on internal compartment loading may be phase-dependent and may involve compensatory loading changes.

Therefore, the primary aim of this study was to quantify the immediate biomechanical effects of short-term gait modifications (toe out, wide stance, and medial thrust) on internal tibiofemoral loading in a varus-aligned mKOA cohort awaiting HTO. Internal compartment forces, pressures, and load distribution were estimated using musculoskeletal modelling (COMAK framework) (Lenhart et al., 2015) to provide mechanistic insight beyond external surrogate metrics and to inform future gait-retraining programmes in this clinical population.

## 2 Methods

### 2.1 Participants

Approval for this work was granted by the Wales Research Ethics Committee 3 (10/MRE09/28) and Cardiff and Vale University Health Board. Written informed consent was obtained from each participant prior to data collection. This was a controlled cohort study. 29 participants (30 knees) with mKOA and varus alignment were recruited from the out-patient clinic of the local senior surgeon (CW) between 2009 and 2020.

The level of mKOA involvement was determined using the Kellgren–Lawrence (KL) (Kellgren and Lawrence, 1957) radiographic score and varus alignment calculated as the mechanical tibiofemoral angle (mTFA) from long leg weight bearing radiographs. Patients were considered eligible for HTO if they presented with symptomatic medial compartment osteoarthritis KL grade 2-3, and grade 4 in selected cases, varus malalignment, and preserved lateral compartment joint space. Patients did not pass initial screening if they were unable to provide informed consent, had neurological or visual conditions affecting movement or a previous injury to the joint under investigation that the treating clinician deemed unsuitable.

### 2.2 Motion analysis

**Fig. 1:**
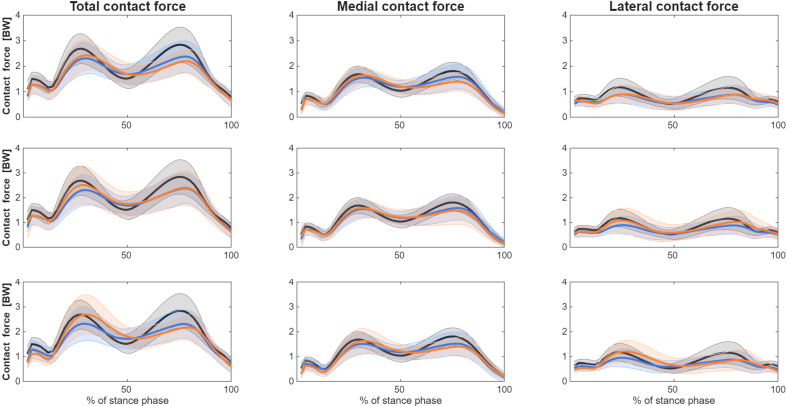
Pre HTO tibiofemoral contact forces under altered gait conditions. The top, middle, and bottom panels correspond to toe out, wide stance, and medial thrust gait, respectively. Traces denote controls (black), pre-HTO normal gait (blue), and the altered gait condition (orange) for each panel.

Three-dimensional gait analysis was performed on patients before (average 1.4 ± 1.4 months) HTO surgery. Gait training and data collection sessions were led by trained researchers with extensive experience in motion analysis and supervised by a motion analysis technician and a qualified nurse. After verbal and visual demonstrations of each gait style and a familiarisation period, participants completed a minimum of three successful trials of unaltered level gait at their self-selected speed. Following these baseline trials, participants performed three additional gait styles: toe out, wide base and medial thrust, within a single visit to the MSK Biomechanics Research Facility (MSKBRF) at Cardiff University. Gait analysis was performed using an 8 or 12 Oqus camera system (Qualisys, Sweden) capturing at 120 Hz, synchronised with either two, four or six (due to laboratory upgrades) force platforms (Bertec Corp., USA) capturing at either 1080 Hz or 2000 Hz. For a visual representation of the marker and lab set up, Figs. 1 and 2 in Whatling et al. (2020) illustrate the same experimental set-up used in the present study, including the modified Cleveland Clinic marker set and the gait analysis data-collection procedures. Markers were placed following a modified Cleveland marker set, as implemented in previous publications (Bowd et al., 2023; Whatling et al., 2020; Whelton et al., 2017).

The adaptations made for each gait style were self-selected but within the boundaries of what would be achievable and tolerated by patients in clinical practice. More specific, a toe out was defined as an increased foot progression angle at initial stance (Whelton et al., 2017). Wide stance gait was defined as an increase in stance width at initial stance (Fregly et al., 2008). Medial thrust gait was deemed to be performed successfully when the participants knee adduction angle during the first half of stance was decreased compared with their unaltered level gait (Gerbrands et al., 2017). These metrics were computed and used to determine the successful completion of each gait style.

### 2.3 Simulation framework

To obtain the tibiofemoral contact forces and pressures, data was processed using a previously validated musculoskeletal modelling workflow (Lenhart et al., 2015). This approach enables estimation of medial and lateral tibiofemoral contact forces and pressures, providing mechanistic insight into compartment-specific loading and potential compensatory shifts that may not be apparent from external moments alone. COMAK model integrates an extended knee model, that allows 6 degrees of freedom (DoF) patellofemoral and tibiofemoral movement, in a generic full-body model (Lenhart et al., 2015). Each leg included 44 musculotendon actuators spanning the hip, knee, and ankle and 14 bundles of non-linear springs that represent the major knee ligaments and posterior capsule.

A non-linear elastic foundation formulation was used to calculate the cartilage contact pressures, based on the penetration depth of the overlapping surface meshes of the contact model (Smith et al., 2018). The cartilage was modelled with a uniformly distributed thickness of 4 mm tibiofemoral and 7 mm patellofemoral thickness (Eckstein et al., 2001; Hudelmaier et al., 2003). The elastic modulus and Poisson's ratio were assumed as 10 MPa and 0.45, respectively (Adouni and Shirazi-Adl, 2014; Li et al., 2001). This model was implemented in SIMM with the Dynamics Pipeline (Musculographics Inc., Santa Rosa, CA) and SD/Fast (Parametric Technology Corp., Needham, MA) to generate the multibody equations of motion.

First, the generic model was scaled to the subjects' anthropometry. Varus alignment was accounted for by implementing the patient-specific mTFA within the original COMAK model, comparable to Van Rossom et al. (2019). To this end, the tibia geometry was rotated in the frontal plane to simulate the malalignment of the affected lower limb. To avoid altering the frontal plane moments by introducing the varus malalignment, the point of force application was expressed in the local foot coordinate system, to make it insensitive to the associated effects of tibia malalignment.

Next, joint angles (pelvic translations and rotations, hip flexion, hip adduction, hip rotation, knee flexion and ankle flexion) were calculated using inverse kinematics. Subsequently, the muscle forces and secondary knee kinematics (11 DoF, i.e., all except knee flexion) required to generate the measured primary hip, knee and ankle accelerations were estimated using the concurrent optimisation of muscle activations and kinematics algorithm. In the optimisation, the weighted sum of squared muscle activations and contact energy were minimised (Smith et al., 2018). As only the knee flexion angle was used in the optimisation, joint kinematics in the secondary knee DoF evolved as a function of muscle, ligament, and contact forces (Lenhart et al., 2015; Smith et al., 2018).

### 2.4 Study statistics

For each trial, the stance phase was identified as the period in which the GRF exceeded 20 N. This study reports parameters averaged over 3 successful trials for each participant.

The magnitude and timing of the first and second peak (FP and SP) of the resultant tibiofemoral contact force was determined during the first and second half of the stance phase, respectively, as well as the minimum force during single leg support (MS). Each variable was determined for the FP, MS, and SP for total knee and the medial and lateral compartments. Each variable was then calculated by an average of three trials. The coinciding average and maximum pressure over the contact surface for the medial, lateral, and total compartments was analysed and included in this paper. Furthermore, the location of the total knee compartment and medial knee compartment centre of pressure (expressed in the local reference frame of the tibia) at FP, SP and MS were analysed and included in this paper.

Paired samples *t*-test was performed in MATLAB (MathWorks, USA) to identify significant differences associated with each gait style, compared to unaltered level gait. Where parametric assumptions were not met, a Wilcoxon signed-rank test was used. Significance was determined when *p* < 0.05 for all statistical tests.

## 3 Results

Participant demographics are outlined in Table 1.

**Table 1:** Demographic and clinical characteristics.

	Number of knees	Gender (M/F)	Age, years Mean (std)	Height, m Mean (std)	Mass, kg Mean (std)	BMI, kg/m^2^ Mean (std)	KL Grade	mTFA (°) Mean (std)
Participants (n=29)	30	25/5	50.70 (8.71)	1.75 (.11)	90.57 (20.17)	29.27 (5.04)	6 KL2; 19 KL3; 5 KL4	7.75 (3.72)

mTFA = varus alignment, calculated as the mechanical tibiofemoral angle (mTFA) from long leg weight bearing radiographs. Positive value for mTFA = varus, std = standard deviation.

During a motion capture session, the lead researcher visually checked whether the correct gait alteration was being achieved and provided verbal feedback to the participants where necessary. The correct implementation of each gait adaptation was defined by the metrics in Table 2. Following post-processing of the motion capture data, medial thrust gait data from ten participants were excluded because they were unable to reduce the maximum knee adduction angle during the first half of stance, indicating that they did not perform an effective medial thrust gait style. Among these ten participants, the maximum knee adduction angle increased by a mean of 1.96° (± 1.71), with individual changes ranging from 0.03° to 2.31°. Therefore, the results in this paper focused on a medial thrust altered gait style refer to *n* = 20 knees. For the wide stance and toe out gait styles *n* = 30 knees, as reported in Table 1.

**Table 2:** Pre-HTO altered gait classifications.

	Stance time (s) Mean (std)	Gait speed (m/s) Mean (std)	FPA (°) Mean (std)	Step width (m) Mean (std)	Maximum knee adduction angle in first half of stance (°) Mean (std)
NL	0.69 (.05)	1.10 (0.24)	16.28 (7.44)	0.16 (0.04)	7.73 (4.52)
TO	0.78 (0.12)	1.09 (0.24)	28.00 (8.14)	N/A	N/A
WS	0.78 (0.13)	1.10 (0.24)	N/A	0.25 (0.07)	N/A
MT	0.81 (0.12)	1.03 (0.22)	N/A	N/A	5.86 (3.97)

NL = normal level gait; TO = toe out gait; WS = wide stance gait; MT = medial thrust gait. Positive foot progression angle (°) = toe out. *n* = 30 for NL, TO, WS; *n* = 20 for MT (10 datasets excluded). std = standard deviation; ° = degree; m = metre; m/s = metres per second; s = seconds.

For the internal joint loading parameters reported below, discrete metrics with corresponding *p*-values are provided in the Supplementary files. Fig. 1 illustrates the total, medial compartment, and lateral compartment tibiofemoral contact forces during toe out (top row), wide-stance (middle row), and medial thrust (bottom row) gait styles.

### 3.1 Toe out altered gait style: Internal joint loading

At FP, adopting a toe out gait pre-HTO significantly increased total, medial, and lateral compartment contact forces and pressures compared to unaltered gait. Medial compartment contact force increased significantly (1.59 BW (0.34) vs 1.70 BW (0.32), *p* = 0.000), while lateral maximum pressure also increased (11.67 MPa (4.69) vs 12.11 MPa (4.37), *p* = 0.048).

At MS, total, medial, and lateral contact forces were significantly unchanged. However, lateral mean (3.08 MPa (1.37) vs 3.37 MPa (1.49), *p* = 0.027) and maximum pressures (6.40 MPa (2.85) vs 7.01 MPa (3.12), *p* = 0.037) increased with toe out gait.

At SP, total and medial contact forces significantly decreased (total: 2.49 BW (0.63) vs 2.31 BW (0.49), *p* = 0.006; medial: 1.63 BW (0.48) vs 1.44 BW (0.37), *p* = 0.000). Medial mean and maximum pressures also reduced significantly (p = 0.006), with no significant changes in the lateral compartment.

Medial-to-total and lateral-to-total contact force ratios were significantly unaffected at FP and MS, but at SP the medial ratio decreased (0.65 (0.12) vs 0.62 (0.11), *p* = 0.002), whilst the lateral ratio increased (0.38 (0.12) vs 0.41 (0.11), *p* = 0.005).

Toe out gait did not alter FP point of application in the medial-lateral direction. At MS, the medial compartment point of application difference between pre-HTO and controls (<1 mm, *p* = 0.045) was eliminated with toe out gait. At SP, both pre-HTO conditions remained more lateral than controls.

Medial contact area significantly increased at FP (223.63 mm^2^ (29.53) vs 226.39 mm^2^ (29.74), *p* = 0.008), was unchanged at MS, and significantly decreased at SP (223.86 mm^2^ (50.52) vs 210.67 mm^2^ (43.85), *p* = 0.004).

### 3.2 Wide stance altered gait style: Internal joint loading

At FP, a wide stance increased total and lateral compartment forces and pressures, as well as medial maximum pressure (increasing from 13.28 to 13.85 MPa, *p* = 0.028). At MS, medial maximum pressure decreased (9.76 vs 9.46 MPa, *p* = 0.014). At SP, medial forces (1.63 to 1.52 BW, *p* = 0.005) and pressures decreased, while lateral forces (0.95 to 1.08 BW, *p* = 0.006) and pressures increased (mean pressure changed from 5.25 to 5.69 MPa, *p* = 0.008; whilst maximum pressure change from 11.33 to 12.17 MPa, *p* = 0.013).

Force ratios shifted accordingly: at FP, the medial-to-total ratio increased (0.64 vs 0.67, *p* = 0.010) and the lateral ratio decreased (0.39 vs 0.36, *p* = 0.011). At SP, the medial ratio decreased (0.65 vs 0.61, *p* = 0.000) and the lateral ratio increased (0.38 vs 0.43, p = 0.000). Point of application shifted laterally at FP (∼1.3 mm, *p* = 0.031) and SP (∼2 mm, p = 0.000). Contact area increased slightly at FP for the total knee (364.3 vs 372.0 mm^2^, *p* = 0.040) and lateral compartment (140.7 vs 148.4 mm^2^, *p* = 0.007), with no significant differences at MS or SP.

### 3.3 Medial thrust altered gait style: Internal joint loading

Despite a significant reduction in gait speed, adopting a medial thrust gait significantly increased total (2.40 to 2.80 BW, *p* < 0.000) and medial (1.59 to 1.67 BW, *p* = 0.018) contact forces in the first half of stance. At SP, no significant differences were observed in medial contact force or contact area.

## 4 Discussion

In this varus-aligned, pre-HTO mKOA cohort, generic gait modifications produced mixed, phase-dependent changes in internal tibiofemoral loading. Toe out and wide stance walking reduced medial compartment loading in late stance but often increased medial forces/pressures in early stance. Medial thrust was difficult for many participants to adopt within a short familiarisation period and, when achieved, was associated with increased early-stance loading. This study is the first to document and quantify the biomechanical effects of three recommended altered gait styles (toe out, wide stance and medial thrust) in individuals with mKOA and varus alignment awaiting HTO.

Only one previous study has assessed altered gait in this specific patient population (Whelton et al., 2017). Key strengths of this study include the evaluation of gait modifications in a varus aligned mKOA cohort awaiting HTO, a population that is clinically important yet underrepresented in the gait retraining literature. In addition, the use of COMAK allowed us to quantify internal medial and lateral compartment contact forces/pressures and load distribution, extending interpretation beyond external surrogate measures and enabling identification of phase-specific trade-offs and compensatory loading. The within-subject design (unaltered vs altered gait within the same participants) further improved sensitivity to detect modest mechanical changes. It is also important to acknowledge that gait alterations in patients with varus-aligned mKOA are multifactorial and may reflect the combined effects of limb alignment, OA severity, symptoms, and prior surgical history. In a recent study of patients eligible for HTO, Valente et al. reported that coexisting factors (OA grade, varus malalignment, and previous meniscectomy) have different impacts on motor function, with varus deformity exerting the primary influence (Valente et al., 2025a). This supports interpreting the present findings largely in the context of varus alignment, while recognising that other patient-specific factors may modulate individual responses to gait retraining.

Using the COMAK framework, toe out gait increased medial compartment contact force at the first peak, indicating higher medial loading in early stance. This is consistent with Legrand et al. (2021), who reported increased external knee adduction moment with toe out gait. Foot progression angle increased by approximately 12° (from 16° to 28°), with substantial inter-individual variability (Jenkyn et al., 2008); however, the therapeutic dose remains unclear. At the second peak, toe out gait reduced medial forces and pressures, shifted the load application laterally, and decreased medial contact area. The clinical relevance of these late-stance changes is hypothesis-generating and requires longer term trials including symptom outcomes; these results also highlight the value of evaluating internal joint mechanics beyond external moments alone.

This is also the first study to evaluate wide stance gait with COMAK in a pre-HTO population. In early stance, wide stance gait increased total, lateral and medial pressures, with medial maximum pressure significantly elevated. By contrast, in late stance, medial forces and pressures decreased, while lateral loading increased. These phase-specific effects suggest potential trade-offs: reduced medial loading during propulsion but increased stresses earlier in stance. Whether the benefits outweigh the drawbacks requires further clinical assessment.

Medial thrust gait proved challenging for this cohort: only 20 of 30 participants achieved the required reduction in peak knee adduction angle. For those who did, internal loading paradoxically increased in early stance, with higher total and compartmental forces and pressures. Short familiarisation may have contributed to exaggerated movement patterns, producing inefficient gait mechanics. Longer-term training may enable patients to adopt a smoother and more effective medial thrust style. These findings highlight the importance of considering feasibility and training requirements when prescribing gait modifications.

The present findings indicate that generic gait modifications can produce mixed, phase-dependent changes in internal loading in a varus, pre-HTO cohort. Although recent trials of personalised foot progression angle retraining have reported improvements in pain alongside biomechanical changes in medial knee OA (e.g., Uhlrich et al., 2025), symptom outcomes were not measured in the current study. Accordingly, we cannot infer symptomatic benefit from the observed loading changes, and the clinical relevance of these phase-specific mechanical effects in a pre-HTO population remains to be established in longer-term trials that include patient-reported outcomes. We have previously reported that HTO surgery realigns the limb, increases gait speed, reduces medial compartment loading in early stance, alters the force application pattern, and decreases medial contact area at 12 months post-surgery (Bowd et al., 2023). Compared with these surgical outcomes, altered gait produced more modest and inconsistent changes, often with opposing effects across stance phases. This suggests that gait modification does not replicate the magnitude or consistency of HTO-related mechanical changes, and its clinical relevance as a pre-surgical adjunct remains uncertain. These findings should also be considered alongside recent evidence that HTO can restore alignment and improve motor function during walking and stair tasks in varus knee OA, with larger and more consistent functional effects than conservative care, although some residual deviations may persist post-operatively (Valente et al., 2025b).

Several limitations should be considered. Participants spanned a wide age range (18–80 years), which does not fully align with the younger-adult rationale and may limit generalisability to a specific age-defined pre-HTO subgroup. There was substantial inter-individual variability in achieving the gait-modification targets, and exclusion of unsuccessful medial thrust trials may bias results toward those able to adopt this strategy. In addition, the musculoskeletal model assumes uniform cartilage thickness; in a cohort with Kellgren-Lawrence grade ≥ 2, this simplification may affect absolute estimates of contact pressure/area, so inference should focus on within-subject, between-condition comparisons.

Although termed unaltered normal level gait, participants had symptomatic mKOA with varus deformity and therefore their baseline gait likely deviated from healthy normative patterns (e.g., reduced speed, altered trunk/hip strategies, and modified frontal-plane mechanics). We did not classify participants according to baseline gait phenotype or severity of compensatory strategies during unaltered walking, and this heterogeneity may have influenced both the ability to achieve the gait-modification targets and the direction/magnitude of loading changes. Future work should stratify or adjust for baseline gait pattern (and other clinical modifiers such as OA severity and prior meniscectomy) to identify whether specific subgroups respond more favourably to a particular gait retraining strategy. Finally, pain and other patient-reported outcomes were not assessed, therefore clinical relevance is hypothesis-generating and requires longer-term trials incorporating symptom outcomes. Future work should stratify or adjust for key clinical modifiers (e.g., OA severity and prior meniscectomy), given their potential to influence motor function and loading responses (Valente et al., 2025a, Valente et al., 2025b).

## 5 Conclusion

This study provides the first evaluation of internal tibiofemoral compartment loading during altered gait styles in patients with varus aligned mKOA awaiting HTO, using musculoskeletal modelling to move beyond external surrogate metrics. Toe out and wide stance gait both reduced medial loading during late stance but increased forces and pressures during early stance, raising concerns about their therapeutic value. Medial thrust gait was difficult to adopt within a short training period and, when performed, increased loading in early stance.

Overall, these results show that small gait modifications can alter tibiofemoral loading patterns, but effects were modest, phase-dependent, and sometimes opposing, with evidence of compensatory loading in some conditions. As pain was not assessed, clinical relevance is hypothesis-generating; longer-term, individualised gait retraining trials incorporating symptom outcomes are required to determine net benefit and assess compensatory loading.

## CRediT authorship contribution statement

**Jake Bowd:** Writing – review & editing, Writing – original draft, Visualization, Validation, Supervision, Software, Resources, Project administration, Methodology, Investigation, Funding acquisition, Formal analysis, Data curation, Conceptualization. **Sam Van Rossom:** Writing – review & editing, Writing – original draft, Supervision, Software, Project administration, Methodology, Formal analysis, Conceptualization. **David W. Elson:** Project administration, Investigation, Data curation, Conceptualization. **Chris Wilson:** Project administration, Investigation, Data curation, Conceptualization. **Ilse Jonkers:** Writing – review & editing, Writing – original draft, Visualization, Validation, Supervision, Software, Resources, Project administration, Methodology, Investigation, Funding acquisition, Formal analysis, Data curation, Conceptualization. **Cathy Holt:** Writing – review & editing, Writing – original draft, Visualization, Validation, Supervision, Software, Resources, Project administration, Methodology, Investigation, Funding acquisition, Formal analysis, Data curation, Conceptualization. **Gemma M. Whatling:** Writing – review & editing, Writing – original draft, Visualization, Validation, Supervision, Software, Resources, Project administration, Methodology, Investigation, Funding acquisition, Formal analysis, Data curation, Conceptualization.

## Ethical approval

Approval for this work was granted by the Wales Research Ethics Committee 3 (10/MRE09/28) and Cardiff and Vale University Health Board.

## Funding

Grant funding received from Arthritis UK (20871 and 18461) and EPSRC (EP/J010111/1).

## Declaration of competing interest

The authors declare that they have no known competing financial interests or personal relationships that could have appeared to influence the work reported in this paper.

## Appendix A Supplementary data

Supplementary material Image 1
