# Supplementary material for: Altered gait strategies show inconsistent medial compartment unloading in varus medial knee osteoarthritis awaiting high tibial osteotomy
Source: Clin Biomech (Bristol). 2026 Jun;136:None. doi: 10.1016/j.clinbiomech.2026.106777 (PMC13100699; doi:10.1016/j.clinbiomech.2026.106777)
Supplement: Supplementary file 1 — Supplementary material [file mmc1.docx]

**Appendix 1** Pre-HTO Altered Gait Internal Knee Joint Loading

|  | | **Pre- HTO NL** | | **Pre- HTO TO** | | **Pre-HTO WS** | | **Pre-HTO MT** | | **Pre NL vs Pre TO** | **Pre NL**  **vs Pre WS** | | **Pre NL**  **vs Pre MT** | |
| --- | --- | --- | --- | --- | --- | --- | --- | --- | --- | --- | --- | --- | --- | --- |
|  | | **Mean (std)** | | **Mean (std)** | | **Mean (std)** | | **Mean (std)** | | **P value** | **P value** | | **P value** | |
| **First peak** | |  | |  | |  | |  | |  |  | |  | |
| **Total knee** | |  | |  | |  | |  | |  |  | |  | |
| Contact force | | 2.40 | | 2.54 | | 2.61 | | 2.80 | |  |  | |  | |
| [BW] | | (0.58) | | (0.55) | | (0.73) | | (0.75) | | **0.002^**^** | **0.002^††^** | | **0.000^**^** | |
| Mean | | 6.10 | | 6.38 | | 6.50 | | 7.01 | |  |  | |  | |
| pressure [MPa] | | (1.66) | | (1.74) | | (2.09) | | (2.14) | | **0.001^**^** | **0.003^††^** | | **0.000^**^** | |
| Max pressure | | 14.14 | | 14.68 | | 15.11 | | 16.23 | |  |  | |  | |
|  | | (4.20) | | (4.51) | | (5.69) | | (5.29) | | **0.042^*^** | **0.024^†^** | | **0.001^**^** | |
| **Medial knee** | |  | |  | |  | |  | |  |  | |  | |
| Contact force | | 1.59 | | 1.70 | | 1.63 | | 1.67 | |  |  | |  | |
| [BW] | | (0.34) | | (0.32) | | (0.36) | | (0.46) | | **0.000^**^** | 0.198 | | **0.018^*^** | |
| Mean | | 6.40 | | 6.79 | | 6.56 | | 6.90 | |  |  | |  | |
| pressure [MPa] | | (1.69) | | (1.84) | | (1.80) | | (2.11) | | **0.000^**^** | 0.102 | | **0.001^††^** | |
| Max pressure | | 13.28 | | 14.31 | | 13.85 | | 14.25 | |  |  | |  | |
|  | | (3.76) | | (4.11) | | (4.10) | | (4.27) | | **0.000^**^** | **0.028^*^** | | **0.002^**^** | |
| **Lateral knee** | |  | |  | |  | |  | |  |  | |  | |
| Contact force | | 0.89 | | 0.92 | | 1.06 | | 1.22 | |  |  | |  | |
| [BW] | | (0.38) | | (0.34) | | (0.52) | | (0.47) | | 0.163 | **0.001^††^** | | **0.000^**^** | |
| Mean | | 5.47 | | 5.63 | | 6.23 | | 7.04 | |  |  | |  | |
| pressure [MPa] | | (2.12) | | (1.98) | | (2.76) | | (2.59) | | 0.158 | **0.001^††^** | | **0.000^**^** | |
| Max pressure | | 11.67 | | 12.11 | | 13.52 | | 15.07 | |  |  | |  | |
|  | | (4.69) | | (4.37) | | (6.37) | | (5.64) | | **0.048^†^** | **0.001^††^** | | **0.000^**^** | |
| **Midstance** | |  | |  | |  | |  | |  |  | |  | |
| **Total knee** | |  | |  | |  | |  | |  |  | |  | |
| Contact force | | 1.36 | | 1.43 | | 1.41 | | 1.49 | |  |  | |  | |
| [BW] | | (0.25) | | (0.36) | | (0.38) | | (0.38) | | 0.198 | 0.642 | | **0.046^*^** | |
| Mean | | 4.39 | | 4.46 | | 4.49 | | 4.74 | |  |  | |  | |
| pressure [MPa] | | (0.82) | | (0.96) | | (0.98) | | (1.05) | | 0.284 | 0.974 | | **0.004^**^** | |
| Max pressure | | 10.01 | | 10.04 | | 10.23 | | 10.99 | |  |  | |  | |
|  | | (2.05) | | (2.35) | | (2.80) | | (2.54) | | 0.627 | 0.294 | | **0.003^††^** | |
| **Medial knee** | |  | |  | |  | |  | |  |  | |  | |
| Contact force | | 1.03 | | 1.05 | | 1.02 | | 1.03 | |  |  | |  | |
| [BW] | | (0.26) | | (0.26) | | (0.29) | | (0.27) | | 0.448 | 0.256 | | 0.411 | |
| Mean | | 4.81 | | 4.85 | | 4.74 | | 4.95 | |  |  | |  | |
| pressure [MPa] | | (0.92) | | (1.02) | | (0.88) | | (1.09) | | 0.593 | 0.117 | | 0.060 | |
| Max pressure | | 9.76 | | 9.64 | | 9.46 | | 10.12 | |  |  | |  | |
|  | | (2.10) | | (2.13) | | (1.96) | | (2.33) | | 0.494 | **0.014^†^** | | **0.040^*^** | |
| **Lateral knee** | |  | |  | |  | |  | |  |  | |  | |
| Contact force | | 0.37 | | 0.43 | | 0.42 | | 0.51 | |  |  | |  | |
| [BW] | | (0.21) | | (0.27) | | (0.35) | | (0.31) | | 0.058 | 0.552 | | **0.026^*^** | |
| Mean | | 3.08 | | 3.37 | | 3.22 | | 3.86 | |  |  | |  | |
| pressure [MPa] | | (1.37) | | (1.49) | | (1.91) | | (1.86) | | **0.027^†^** | 0.991 | | **0.019^*^** | |
| Max pressure | | 6.40 | | 7.01 | | 6.78 | | 8.05 | |  |  | |  | |
|  | | (2.85) | | (3.12) | | (4.24) | | (3.96) | | **0.037^†^** | 0.837 | | **0.024^*^** | |
| **Second peak** | |  | |  | |  | |  | |  |  | |  | |
| **Total knee** | |  | |  | |  | |  | |  |  | |  | |
| Contact force | | 2.49 | | 2.31 | | 2.51 | | 2.29 | |  |  | |  | |
| [BW] | | (0.63) | | (0.49) | | (0.68) | | (0.56) | | **0.006^**^** | 0.417 | | 0.271 | |
| Mean | | 6.02 | | 5.85 | | 6.06 | | 5.90 | |  |  | |  | |
| pressure [MPa] | | (1.07) | | (0.98) | | (1.19) | | (1.62) | | 0.071 | 0.417 | | 0.147 | |
| Max pressure | | 14.19  (3.06) | | 13.70  (3.06) | | 14.33  (4.05) | | 14.05  (4.91) | | 0.226 | | 0.469 | 0.260 | |
| **Medial knee** | |  | |  | |  | |  | |  | |  |  | |
| Contact force [BW] | | 1.63  (0.48) | | 1.44  (0.37) | | 1.52  (0.53) | | 1.45 (0.37) | | **0.000^††^** | | **0.005^†^** | 0.084 | |
| Mean pressure [MPa] | | 6.44  (1.37) | | 6.11  (1.24) | | 6.15  (1.37) | | 6.06 (1.69) | | **0.006^**^** | | **0.002^*^** | 0.070 | |
| Max pressure | | 13.18  (2.87) | | 12.36  (2.68) | | 12.58  (2.82) | | 12.36 (3.83) | | **0.004^**^** | | **0.003^*^** | **0.049^†^** | |
| **Lateral knee** | |  | |  | |  | |  | |  | |  |  | |
| Contact force [BW] | | 0.95  (0.37) | | 0.95  (0.34) | | 1.08  (0.46) | | 0.93 (0.45) | | 0.905 | | **0.006^††^** | 0.546 | |
| Mean pressure [MPa] | | 5.25  (1.46) | | 5.37  (1.54) | | 5.69  (1.83) | | 5.44 (2.34) | | 0.133 | | **0.008^††^** | 0.809 | |
| Max pressure | | 11.33  (3.58) | | 11.54  (3.66) | | 12.17 (4.54) | | 11.67 (5.40) | | 0.230 | | **0.013^†^** | 0.841 | |

Significant difference (p < 0.01) indicated by ** where parametric or †† where non-parametric tests used. TO = toe out gait; WS = wide stance gait; MT = medial thrust gait. BW = body weight; MPa = megapascals.

**Appendix 2** Pre-HTO Altered Gait Contact Force Ratios

|  | **Pre-HTO NL** | **Pre-HTO TO** | **Pre-HTO WS** | **Pre-HTO MT** | **Pre NL vs Pre TO** | **Pre NL vs Pre WS** | **Pre NL vs Pre MT** |
| --- | --- | --- | --- | --- | --- | --- | --- |
|  | **Mean (std)** | **Mean (std)** | **Mean (std)** | **Mean (std)** | **P value** | **P value** | **P value** |
|  |  |  |  |  |  |  |  |
| **First peak** |  |  |  |  |  |  |  |
| MED / TOTAL | 0.67 (0.10) | 0.68 (0.08) | 0.64 (0.10) | 0.60 (0.09) | 0.708 | **0.010^**^** | **0.005^**^** |
| LAT / TOTAL | 0.36 (0.10) | 0.35 (0.08) | 0.39 (0.10) | 0.43 (0.10) | 0.643 | **0.011^**^** | **0.008^**^** |
|  |  |  |  |  |  |  |  |
| **Midstance** |  |  |  |  |  |  |  |
| MED / TOTAL | 0.76 (0.14) | 0.74 (0.13) | 0.74 (0.17) | 0.70 (0.15) | 0.149 | 0.325 | 0.097 |
| LAT / TOTAL | 0.27 (0.15) | 0.29 (0.14) | 0.28 (0.18) | 0.33 (0.16) | 0.153 | 0.443 | 0.131 |
|  |  |  |  |  |  |  |  |
| **Second peak** |  |  |  |  |  |  |  |
| MED / TOTAL | 0.65 (0.12) | 0.62 (0.11) | 0.61 (0.13) | 0.64 (0.12) | **0.002^**^** | **0.000^††^** | 0.920 |
| LAT / TOTAL | 0.38 (0.12) | 0.41 (0.11) | 0.43 (0.14) | 0.39 (0.13) | **0.005^**^** | **0.000^††^** | 0.910 |

Significant difference (p<0.01) indicated by ** where parametric or †† where non-parametric tests used. Significant difference (p < 0.01) indicated by ** where parametric or †† where non- parametric tests used. TO = toe out gait; WS = wide stance gait; MT = medial thrust gait. BW = body weight; MPa = megapascals. MED = medial compartment contact force; LAT = lateral compartment contact force; TOTAL = total tibiofemoral contact force.

**Appendix 3** Pre-HTO Altered Gait Point of Application

|  | **Pre-HTO NL** | **Pre-HTO TO** | **Pre-HTO WS** | **Pre-HTO MT** | **Pre NL vs Pre TO** | **Pre NL vs Pre WS** | **Pre NL vs Pre MT** |
| --- | --- | --- | --- | --- | --- | --- | --- |
| **mm** | **Mean (std)** | **Mean (std)** | **Mean (std)** | **Mean (std)** | **P value** | **P value** | **P value** |
| **First peak** |  |  |  |  |  |  |  |
| **Total knee** |  |  |  |  |  |  |  |
| Anterior (+) |  |  |  |  |  |  |  |
| / posterior (-) | -2.07 (2.98) | -2.45 (2.98) | -2.43 (3.26) | -3.78 (3.08) | **0.040^*^** | 0.064 | **0.000^**^** |
| Lateral (+) / |  |  |  |  |  |  |  |
| medial (-) | -4.91 (4.19) | -5.00 (3.42) | -3.67 (4.42) | -2.23  (3.44) | 0.782 | **0.031^*^** | **0.011^*^** |
| **Medial knee** |  |  |  |  |  |  |  |
| Anterior (+) |  |  |  |  |  |  |  |
| / posterior (-) | -0.30 (3.27) | -0.97 (3.30) | -0.61 (3.46) | -1.66  (3.09) | **0.030^†^** | 0.403 | **0.001^††^** |
| Lateral (+) / |  |  |  |  |  |  |  |
| medial (-) | -18.08 (1.52) | -18.14 (1.65) | -18.15 (1.74) | -18.37  (1.90) | 0.170 | 0.565 | 0.141 |
| **Lateral knee** |  |  |  |  |  |  |  |
| Anterior (+) |  |  |  |  |  |  |  |
| / posterior (-) | -5.12 (2.50) | -5.19 (2.41) | -5.39 (2.76) | -6.67  (2.52) | 0.623 | 0.103 | **0.007^**^** |
| Lateral (+) / |  |  |  |  |  |  |  |
| medial (-) | 19.69 (2.14) | 19.89 (2.11) | 19.80 (2.29) | 20.35  (2.07) | 0.245 | 0.637 | 0.104 |
| **Midstance** |  |  |  |  |  |  |  |
| **Total knee** |  |  |  |  |  |  |  |
| Anterior (+) |  |  |  |  |  |  |  |
| / posterior (-) | 4.07 (2.26) | 3.82 (3.07) | 3.69 (3.05) | 3.72  (3.97) | 0.530 | 0.754 | 0.763 |
| Lateral (+) / |  |  |  |  |  |  |  |
| medial (-) | -7.53 (7.14) | -6.98 (5.97) | -7.28 (7.98) | -4.89  (6.69) | 0.277 | 0.496 | 0.125 |
| **Medial knee** |  |  |  |  |  |  |  |
| Anterior (+) |  |  |  |  |  |  |  |
| / posterior (-) | 6.42 (2.69) | 6.09 (3.07) | 5.83 (3.02) | 6.49  (3.33) | 0.820 | 0.174 | 0.899 |
| Lateral (+) / |  |  |  |  |  |  |  |
| medial (-) | -17.09 (2.14) | -17.13 (1.75) | -17.29 (2.15) | -16.80  (1.88) | 0.794 | 0.300 | 0.752 |
| **Lateral knee** |  |  |  |  |  |  |  |
| Anterior (+) |  |  |  |  |  |  |  |
| / posterior (-) | -2.18 (2.21) | -2.39 (2.36) | -1.83 (2.53) | -2.55  (3.55) | 0.336 | 0.256 | 0.955 |
| Lateral (+) / |  |  |  |  |  |  |  |
| medial (-) | 18.91 (3.55) | 19.09 (3.21) | 18.41 (5.42) | 19.91  (4.70) | 0.462 | 0.673 | 0.212 |
| **Second peak** |  |  |  |  |  |  |  |
| **Total knee** |  |  |  |  |  |  |  |
| Anterior (+) |  |  |  |  |  |  |  |
| / posterior (-) | 4.45 (3.36) | 3.68 (4.12) | 3.89 (3.99) | 2.50  (4.43) | 0.078 | 0.090 | **0.035^*^** |
| Lateral (+) / |  |  |  |  |  |  |  |
| medial (-) | -2.59 (5.13) | -1.43 (4.70) | -0.62 (5.84) | -2.72  (4.87) | **0.002^**^** | **0.000^††^** | 0.419 |
| **Medial knee** |  |  |  |  |  |  |  |
| Anterior (+) |  |  |  |  |  |  |  |
| / posterior (-) | 8.15 (3.38) | 7.53 (4.37) | 7.53 (4.16) | 6.22  (3.86) | 0.275 | 0.084 | **0.029^*^** |
| Lateral (+) / |  |  |  |  |  |  |  |
| medial (-) | -15.83 (1.76) | -15.88 (1.65) | -15.68 (1.93) | -16.48  (2.11) | 0.738 | 0.524 | **0.025^*^** |
| **Lateral knee** |  |  |  |  |  |  |  |
| Anterior (+) |  |  |  |  |  |  |  |
| / posterior (-) | -2.59 (3.39) | -2.72 (3.37) | -1.97 (3.73) | -4.25  (3.17) | 0.417 | **0.045^†^** | **0.040^†^** |
| Lateral (+) / |  |  |  |  |  |  |  |
| medial (-) | 19.48 (3.40) | 20.37 (3.03) | 20.26 (3.38) | 19.51  (2.89) | **0.007**** | **0.016^**^** | 0.711 |

Significant difference (p < 0.01) indicated by ** where parametric or †† where non-parametric tests used. TO = toe out gait; WS = wide stance gait; MT = medial thrust gait. std = standard deviation. mm = millimetres. X = anterior; Z = lateral. COP = centre of pressure

**Appendix 4** Pre-HTO Altered Gait Knee Contact Area

|  | **Pre-HTO NL** | **Pre-HTO TO** | **Pre-HTO WS** | **Pre-HTO MT** | **Pre NL vs Pre TO** | **Pre NL vs Pre WS** | **Pre NL vs Pre MT** |
| --- | --- | --- | --- | --- | --- | --- | --- |
| **mm^2^** | **Mean (std)** | **Mean (std)** | **Mean (std)** | **Mean (std)** | **P value** | **P value** | **P value** |
| **First peak** |  |  |  |  |  |  |  |
| Total | 364.30  (49.61) | 369.79  (46.44) | 372.04 (47.95) | 371.84  (44.59) | 0.055 | **0.040^*^** | 0.198 |
| Medial | 223.63  (29.53) | 226.39  (29.74) | 223.69 (29.87) | 217.70  (20.86) | **0.008^††^** | 0.978 | 0.184 |
| Lateral | 140.68  (28.69) | 143.40  (25.71) | 148.35 (28.96) | 154.13  (27.91) | 0.194 | **0.007^*^** | **0.032^*^** |
| **Midstance** |  |  |  |  |  |  |  |
| Total | 286.65  (55.88) | 295.92  (59.84) | 287.79 (62.65) | 291.61  (59.81) | 0.102 | 0.837 | 0.744 |
| Medial | 189.50  (36.97) | 192.73  (36.07) | 189.90 (35.87) | 185.14  (33.83) | 0.417 | 0.642 | 0.856 |
| Lateral | 97.16  (35.50) | 103.20  (34.21) | 97.90 (45.34) | 106.46  (36.11) | 0.095 | 0.957 | 0.459 |
| **Second peak** |  |  |  |  |  |  |  |
| Total | 383.65  (91.11) | 367.38  (76.97) | 384.70 (87.65) | 367.57  (75.74) | **0.018^*^** | 0.905 | 0.272 |
| Medial | 223.86  (50.52) | 210.67  (43.85) | 216.92 (50.99) | 217.95  (36.90) | **0.004^**^** | 0.071 | 0.674 |
| Lateral | 159.80  (48.03) | 156.72  (39.22) | 167.77 (45.71) | 149.62  (44.11) | 0.329 | 0.112 | 0.097 |

Significant difference (p < 0.01) indicated by ** where parametric or †† where non- parametric tests used. TO = toe out gait; WS = wide stance gait; MT = medial thrust gait. std = standard deviation. mm^2^ = millimetres squared
